# Supplementary material for: Next generation insect taxonomic classification by comparing different deep learning algorithms
Source: PLoS One. 2022 Dec 30;17(12):e0279094. doi: 10.1371/journal.pone.0279094 (PMC9803097; doi:10.1371/journal.pone.0279094)
Supplement: Attachment — Submitted filename: Rebuttal letter.docx [file pone.0279094.s001.docx]

Dear Editors,

We thank the editors and reviewers for your time and generous comments on the manuscripts and we have revised the manuscript to address your concerns and to meet with the journal requirements.

We have included the point-to-point response to reviewer as below and attached both ‘Revised Manuscript with Track Changes' and ‘Manuscript’ in the system.

We believe the manuscript’s quality is now improved and appropriate for publication in PLOS One

Thanks again,

Your sincerely,

Song-Quan Ong

**Point-by-point response**

**Journal Requirements:**

1. Please ensure that your manuscript meets PLOS ONE's style requirements, including those for file naming. The PLOS ONE style templates can be found at <https://journals.plos.org/plosone/s/file?id=wjVg/PLOSOne_formatting_sample_main_body.pdf> and <https://journals.plos.org/plosone/s/file?id=ba62/PLOSOne_formatting_sample_title_authors_affiliations.pdf>

The manuscript has been revised to meet the PLOS ONE's style requirements

2. Please review your reference list to ensure that it is complete and correct. If you have cited papers that have been retracted, please include the rationale for doing so in the manuscript text or remove these references and replace them with relevant current references. Any changes to the reference list should be mentioned in the rebuttal letter that accompanies your revised manuscript. If you need to cite a retracted article, indicate the article’s retracted status in the References list and also include a citation and full reference for the retraction notice.

The reference list has been reviewed and correct according to PLOS One style. In order to address the comments of Reviewer 2 about the data augmentation, we added one reference [10] –

Shorten C, Khoshgoftaar TM. A survey on image data augmentation for deep learning. Journal of Big Data. 2019;6(1):1-48.

To address the comments of Reviewer 1 about references should be recent.

We also replaced one reference [13] (previous version is [12]) into:

Tang L, Zhang H, Zhang B. A note on error bars as a graphical representation of the variability of data in biomedical research: choosing between standard deviation and standard error of the mean. Journal of Pancreatology. 2019;2(03):69-71.

3. We noticed you have some minor occurrence of overlapping text with the following previous publication(s), which needs to be addressed:

- <https://onlinelibrary.wiley.com/doi/10.1002/ps.7028>

The text that needs to be addressed involves the Introduction and the Results sections.

In your revision ensure you cite all your sources (including your own works), and quote or rephrase any duplicated text outside the methods section. Further consideration is dependent on these concerns being addressed.

The content of the occurrence of overlapping has been rewritten.

**Reviewer #1:**

The authors have proposed a novel algorithm version of Insect Taxonomic Classification using CNN. The following are the comments that needs to addressed in the manuscript

- Abstract and conclusion needs the accuracy/ performance evaluation results to be specified.

Thank you for your comment. The performance evaluation results of F1-score for InceptionV3 has been added in the abstract as the sentences of "The InceptionV3 model has advantages over other models due to its high performance in distinguishing insect order and family, which is having F1-score of 0.75 and 0.79, respectively"

- The research gap and the proposed solution should be highlighted before the methodology

Thank you for your comment. Research gap and hypothesis were added in the end of introduction before the methodology in line 82 - 95.

"However, most of these previous studies of DL models on insect classification were not designed to assess the capability of DL in classifying different taxonomic levels. For instance, research questions such as “What will the performance of a DL model be as the taxonomic level decreases?” and “Will a single DL architecture be sufficient to classify specimens regardless of their taxonomic levels?” remain. Since previous studies assumed that insect classification can be done according to the concept of one- size-fits-all, the most appropriate algorithm could be the solution for most classifications at the taxonomic level. We hypothesise that different algorithms for classification are needed for different taxonomic levels, because the lower the level, the closer the external morphology. For this reason, this study aims to evaluate the ability of DL models in classifying insect specimens at different taxonomic levels. We compared the performances of four DL models, InceptionV3, VGG19, MobileNetV2, and Xception, in classifying three taxonomic levels: order, family, and genus."

- The novelty of the proposed work should be highlighted.

We have restructured the sentences and emphasis of the novelty of the study, which are

1. Customised datasets (line 191 to 193)

2. No one-size-fits-all model, and each taxa levels is having their own best performed algorithm (line 221)

- Is there any open-source database available for for this application? is yes then the results should be obtained the the same and reported in the article.

Yes, there is a open source of dataset available in [15]. We have mentioned the dataset in line 197 and data availability.

- Discussion part needs to be elaborated and how the proposed method is efficient compared to other existing algorithms

Thank you for your comment. We elaborated how our result is more effective compared to other studies in line 281-283, where describing our result is more comprehensive and having better performance coverage including the F1-score and precision.

- references should be recent (less than 5-7 years)

Thank you for your comment. We updated the reference [12] (the one reference with older than 7 years) into:

Tang L, Zhang H, Zhang B. A note on error bars as a graphical representation of the variability of data in biomedical research: choosing between standard deviation and standard error of the mean. Journal of Pancreatology. 2019 Sep 1;2(03):69-71.

Which published in 2019 and having more compherasive discussion on the error bar that we used as the stat tool in this study.

**Reviewer #2:**

In this work, the authors study the classification performance of four deep CNN models (InceptionV3, VGG19, MobileNetV2 and Xception) in classifying insect images into three taxonomic levels (order, family and genus). I have only a few minor comments to improve the paper:

1. The introduction section could elaborate on the motivation for the work.

Thank you for your comment. Motivation, research gap and hypothesis were added in the end of introduction in line 82 - 95.

"However, most of these previous studies of DL models on insect classification were not designed to assess the capability of DL in classifying different taxonomic levels. For instance, research questions such as “What will the performance of a DL model be as the taxonomic level decreases?” and “Will a single DL architecture be sufficient to classify specimens regardless of their taxonomic levels?” remain. Since previous studies assumed that insect classification can be done according to the concept of one- size-fits-all, the most appropriate algorithm could be the solution for most classifications at the taxonomic level. We hypothesise that different algorithms for classification are needed for different taxonomic levels, because the lower the level, the closer the external morphology. For this reason, this study aims to evaluate the ability of DL models in classifying insect specimens at different taxonomic levels. We compared the performances of four DL models, InceptionV3, VGG19, MobileNetV2, and Xception, in classifying three taxonomic levels: order, family, and genus."

2. The authors propose that the classification pipeline must include several classification algorithms for different taxonomic ranks. It will be interesting if the authors could elaborate on the characteristic that the classification algorithm should possess to perform remarkably for each taxonomic rank.

Thank you for your comment. We elaborate more on the algorithm characteristic in the section of discussion, where taking note of the model characteristic such as trainable parameters versus the taxonomic level, which a decrease of parameters (VGG19 to MobileNetV2), higher the performance with lower taxonomic levels.

3. How did the authors choose the optimal hyperparameters for the model?

The optimal hyperparameters were chosen manually by comparing different learning rate and two of standard optimisers. We have described the process of studying the optimization of model in line 156-159 "This study trained deep learning neural networks by using the adaptive learning rate optimization (Adam) algorithm with learning rate hyperparameters of 0.001, 0.0001, and 0.00001 to control the rate of change of the model during each step of the optimization process.

4. Even though 2000 images may not be sufficient, it might be interesting to see how the model performs on the original dataset of ~2000 images and compare the performance with the dataset that had the rotated images as well.

Thank you for your comment. Comparison of model performance by using original image number and data augmented number is not the objective of this study, therefore we added one reference [10] to justify the needs of augmenting the data before the deep model development.

Shorten C, Khoshgoftaar TM. A survey on image data augmentation for deep learning. Journal of big data. 2019 Dec;6(1):1-48.

5. The authors also fixed the number of training epochs at 100, which might quite low. The authors might consider increasing the number of training epochs and evaluating the performance.

We applied early stop mechanism (Appendices II and III) to prevent the overfitting for the image classification. In other words, higher epochs could lead to the issue of overfitting, we further justified the epochs number with an additional reference - A survey on Image Data Augmentation for Deep Learning (especially Fig 1)

Shorten C, Khoshgoftaar TM. A survey on image data augmentation for deep learning. Journal of big data. 2019 Dec;6(1):1-48

**Reviewer #3**:

The paper addresses the class classification task according to the taxonomic ranks of insects—order, family, and genus and compared the generalization of four state-of-the-art deep convolutional neural network (DCNN) architectures. The statistical analysis for all the four Deep learning models with respect to taxonomy levels are showcased. Model classification based on the individual group are also well depicted.

Concern: Little more detailing on preprocessing of the data and the InceptionV3 model layers could be added relevance

Thank you for your comment. We added more details on the preprocessing of data in line 140-141 "The base images (0 degrees, without rotation) and all the rotated images (90, 180, and 270 degrees) used for training are not used for the testing and validation sets.", and InceptionV3 model layers in line 226 - "For instance, the VGG19 model performed the best for order, InceptionV3 performed the best for family, and MobileNetV2 performed the best for genus. The inceptionV3 that having a total of 42 layers is having advantages of consistent performance from one level to another, which did not perform significantly differently when the taxonomic level was lowered from order to family, in contrast with other models that exhibited significantly lower performance when the level was lower.

Thank you very much for the valuable feedback and comment.

Best regards,

Song-Quan Ong
